# Editorial: Advanced integration of large language models for autonomous systems and critical decision support

**DOI:** 10.3389/frai.2026.1962295

**Published:** 2026-09-07

**Authors:** I. de Zarzà, J. de Curtò, Carlos T. Calafate

**Affiliations:** 1 Human-Centered AI, Data and Software, Luxembourg Institute of Science and Technology, Esch-sur-Alzette, Luxembourg; 2 Department of Computer Applications in Science and Engineering, Barcelona Supercomputing Center, Barcelona, Spain; 3 Escuela Técnica Superior de Ingeniería (ICAI), Universidad Pontificia Comillas, Madrid, Spain; 4 Departamento de Informática de Sistemas y Computadores, Universitat Politècnica de València, Valencia, Spain

**Keywords:** AI governance, autonomous systems, critical decision support, human oversight, large language models, multi-agent systems, safety assurance, semantic grounding

## 1 Motivation: from capable models to dependable systems

Large language models (LLMs) have shown transformative potential in autonomous systems and critical decision-making, yet standalone models remain limited in robustness, reliability, and safety assurance when deployed in high-stakes environments. This Research Topic began from the premise that such limitations are better addressed by structured integration of multiple specialized models than by scaling any single one (Guo et al., 2024). We invited work on multi-LLM integration for perception, navigation, and decision-making in robots, drones, and vehicles; on human–robot collaboration; on high-stakes decision support; on verification, uncertainty quantification, and safety assurance; and on real-time adaptation. Seven contributions were accepted, spanning agent generation, orchestration, perception, query translation, automated machine learning, intrusion detection, and governance.

Deployment in these settings changes the central question. Performance can no longer be judged by fluency or task accuracy alone; it must also be assessed through grounding, reproducibility, latency, calibration, failure containment, human oversight, and auditability. Across domains, the contributions converge on a common conclusion: dependable autonomy is principally a systems-engineering problem. Reliability emerges not from trusting a single model, but from structuring how models are composed, constrained, checked, and connected to action.

## 2 Orchestration and adaptive composition

Perera et al. challenge the fixed-team assumption of many multi-agent systems. Their Initial Automatic and Dynamic Real-Time Agent Generation mechanisms create specialized agents from evolving conversational context. In the evaluated medical scenario, dynamic generation improved coverage, lexical diversity, and thematic relevance over a static AutoGen configuration, treating system composition itself as an adaptive variable. The same move from fixed programs toward prompt-defined behavior appears in LLM-driven swarm simulations (Jimenez-Romero et al., 2025).

Zhou and Chan address the complementary problem of reproducibility. Their orchestrator, ORCH, decomposes a problem, gathers analyses from heterogeneous models, and merges them through a deterministic protocol; an optional exponential moving average module adapts routing from historical feedback. Gains are strongest on harder reasoning tasks, but entail substantial latency and cost. Taken together, these studies show that adaptation and determinism are not opposites: agent membership and routing may change, while interfaces, fallback rules, and aggregation procedures remain explicit and auditable, as in ensemble-and-arbiter designs where inter-model disagreement is measured and routed to human review (Lipianina-Honcharenko et al., 2026).

## 3 Grounded decision support in real environments

Nazar et al. move orchestration into a safety-relevant physical context. Assuming distraction has already been detected, the system combines camera and GPS inputs with specialized agents for visual perception, speed limits, traffic, and weather, and then generates concise verbal alerts, reporting approximately 86% semantic intervention correctness at an average latency of 1.74 s. Modular fusion can thus support real-time, context-aware intervention, though deployment depends on robust sensing, connectivity, and control of response verbosity.

Huang and Tao apply the same integrative logic to the machine-learning lifecycle. Their agent connects natural-language interaction to preprocessing, task inference, model selection, hyperparameter optimization, and evaluation. It highlights how LLMs lower technical barriers (Yao et al., 2025), but also exposes production concerns that recur across the collection: generated-code validation, reproducibility, privacy, model bias, API cost, and failure recovery.

Two studies focus directly on critical infrastructure. Dranca et al. combine a knowledge graph (Mariotti et al., 2024), entity linking (Mavridis et al., 2025), compact fine-tuned models, and syntactic and semantic validation to translate Spanish questions into Flux and InfluxQL queries over a live agro-food plant database. Their three-layer evaluation (high parser validity, moderate execution success, and lower result correctness) locates the remaining bottleneck not in surface syntax but in semantic grounding. Saidi also combines representations rather than relying on generation alone: in NIDS-β^*^, a compact Transformer encoder for flow sequences is fused with tabular features, supervised and one-class heads, and attention- and SHAP-based explanations (Mohale and Obagbuwa, 2025), reaching an *F*_1_-score of 0.972 on zero-day traffic and an expected calibration error of 1.9%. Calibration determines whether confidence can safely trigger escalation. Together, they show that critical decision support requires explicit domain representations, calibrated outputs, and verification against the operational environment (Wickramasinghe Brahmana et al., 2025).

## 4 Governance as an architectural property

Calboreanu makes the architectural argument most explicit. LATTICE separates planning, execution, and governance so that no component both decides an action and judges compliance; it applies policy-as-code through gated execution, escalates uncertain cases to human operators, and preserves provenance through cryptographic audit trails. The evaluation is deliberately planner-invariant: across four frontier planner families, a confidence-threshold baseline's false-allow rate ranged from 0.03 to 0.998, whereas the governed implementation admitted no unsafe actions at a conservative operating point that auto-allowed none. The study frames this as a safety and authorization analysis rather than an autonomy benchmark, with claims conditional on stated trusted-infrastructure assumptions. This distinction matters: model competence and authorization assurance are related but distinct engineering objectives. Related policy-enforced intrusion-detection architectures similarly externalize governance into an auditable policy layer (Adabara et al., 2026).

## 5 What the contributions establish together

Four design principles connect the seven articles. First, specialization helps only when coordination is designed; more agents do not automatically improve a system. Second, free-form output should become inspectable intermediate objects: roles, subproblems, schema entities, queries, scores, policies, or action bundles. Compiling LLM narratives into parameterized cooperation policies reaches the same conclusion: structured representations make interventions attributable, constrainable, and governable (de Curtò et al., 2026). Third, validation must occur at system boundaries (Anh-Hoang et al., 2025), before generated text becomes a database query, driver alert, security response, or executable action. Fourth, evaluation must move beyond aggregate accuracy (Polonioli, 2025) to include latency, calibration, false alarms, abstention or escalation, resource cost, robustness under shift, and the workload imposed on human operators.

The next phase should connect these principles into end-to-end assurance cases. It requires interoperable agent–tool contracts, benchmarks covering distribution shift and adversarial faults (Radanliev et al., 2026), selective autonomy with tested fallback behavior, and human-centered studies of explanation and escalation. The central lesson is measured but consequential: LLMs become suitable for autonomous systems and critical decision support not as self-sufficient decision makers, but when embedded within architectures that make uncertainty visible, constrain action, preserve accountability, and retain meaningful human control (Santoni de Sio and van den Hoven, 2018).

We thank all contributing authors, reviewers, and the Frontiers editorial team for advancing this interdisciplinary discussion.

## References

[B1] Adabara I. Sadiq B. O. Shuaibu A. N. Danjuma Y. I. Maninti V. Joe M. (2026). The Agentic AI Framework (AAIF): a policy-enforced architecture for accountable and high-performance intrusion detection. Front. Artif. Intell. 9:1755696. doi: 10.3389/frai.2026.1755696 42325280 PMC13275711

[B2] Anh-Hoang D. Tran V. Nguyen L.-M. (2025). Survey and analysis of hallucinations in large language models: attribution to prompting strategies or model behavior. Front. Artif. Intell. 8:1622292. doi: 10.3389/frai.2025.1622292 41098969 PMC12518350

[B3] de Curtò J. de Zarzà I. Cabot J. Manzoni P. Calafate C. T. (2026). From LLM narratives to parameterized cooperation policies in multi-agent systems. Front. Artif. Intell. 9:1820827. doi: 10.3389/frai.2026.1820827 42499760 PMC13395858

[B4] Guo T. Chen X. Wang Y. Chang R. Pei S. Chawla N. V. . (2024). “Large language model based multi-agents: a survey of progress and challenges,” in Proceedings of the thirty-third international joint conference on artificial intelligence (Jeju), 8048–8057.

[B5] Jimenez-Romero C. Yegenoglu A. Blum C. (2025). Multi-agent systems powered by large language models: applications in swarm intelligence. Front. Artif. Intell. 8:1593017. doi: 10.3389/frai.2025.1593017 40469074 PMC12135685

[B6] Lipianina-Honcharenko K. Drakokhrust T. Bykovyy P. Turchynov K. Ihnatiev I. (2026). Bridging jurisdictions and legislatures: an LLM ensemble and arbiter framework for automated legal risk triage in digital media. Front. Artif. Intell. 9:1841104. doi: 10.3389/frai.2026.1841104 42466202 PMC13372744

[B7] Mariotti L. Guidetti V. Mandreoli F. Belli A. Lombardi P. (2024). Combining large language models with enterprise knowledge graphs: a perspective on enhanced natural language understanding. Front. Artif. Intell. 7:1460065. doi: 10.3389/frai.2024.1460065 39258232 PMC11385612

[B8] Mavridis A. Tegos S. Anastasiou C. Papoutsoglou M. Meditskos G. (2025). Large language models for intelligent RDF knowledge graph construction: results from medical ontology mapping. Front. Artif. Intell. 8:1546179. doi: 10.3389/frai.2025.1546179 40352975 PMC12061982

[B9] Mohale V. Z. Obagbuwa I. C. (2025). A systematic review on the integration of explainable artificial intelligence in intrusion detection systems to enhancing transparency and interpretability in cybersecurity. Front. Artif. Intell. 8:1526221. doi: 10.3389/frai.2025.1526221 40040929 PMC11877648

[B10] Polonioli A. (2025). Moving LLM evaluation forward: lessons from human judgment research. Front. Artif. Intell. 8:1592399. doi: 10.3389/frai.2025.1592399 40495932 PMC12149859

[B11] Radanliev P. Santos O. Maple C. (2026). Threats and vulnerabilities in artificial intelligence and agentic AI models. Front. Artif. Intell. 9:1731566. doi: 10.3389/frai.2026.1731566 41766945 PMC12947123

[B12] Santoni de Sio F. van den Hoven J. (2018). Meaningful human control over autonomous systems: a philosophical account. Front. Robot. AI 5:15. doi: 10.3389/frobt.2018.00015 33500902 PMC7806098

[B13] Wickramasinghe Brahmana C. S. Marino D. De Silva D. Manic M. (2025). Editorial: Machine learning for cybersecurity. Front. Artif. Intell. 8:1640609. doi: 10.3389/frai.2025.1640609 41098968 PMC12518219

[B14] Yao J. Zhang L. Huang J. (2025). Evaluation of large language model-driven AutoML in data and model management from human-centered perspective. Front. Artif. Intell. 8:1590105. doi: 10.3389/frai.2025.1590105 40838130 PMC12362983

